# Epigenetic Inheritance: Concepts, Mechanisms and Perspectives

**DOI:** 10.3389/fnmol.2018.00292

**Published:** 2018-09-28

**Authors:** Irene Lacal, Rossella Ventura

**Affiliations:** ^1^Department of Physiology and Pharmacology, Sapienza University of Rome, Rome, Italy; ^2^Department of Psychology and “Daniel Bovet” Center, Sapienza University of Rome, Rome, Italy; ^3^Fondazione Santa Lucia, IRCCS, Rome, Italy

**Keywords:** transgenerational, epigenetic, inheritance, transmission, psychopathology, stress, microRNA, methylation

## Abstract

Parents’ stressful experiences can influence an offspring’s vulnerability to many pathological conditions, including psychopathologies, and their effects may even endure for several generations. Nevertheless, the cause of this phenomenon has not been determined, and only recently have scientists turned to epigenetics to answer this question. There is extensive literature on epigenetics, but no consensus exists with regard to how and what can (and must) be considered to study and define epigenetics processes and their inheritance. In this work, we aimed to clarify and systematize these concepts. To this end, we analyzed the dynamics of epigenetic changes over time in detail and defined three types of epigenetics: a direct form of epigenetics (DE) and two indirect epigenetic processes—within (WIE) and across (AIE). DE refers to changes that occur in the lifespan of an individual, due to direct experiences with his environment. WIE concerns changes that occur inside of the womb, due to events during gestation. Finally, AIE defines changes that affect the individual’s predecessors (parents, grandparents, etc.), due to events that occur even long before conception and that are somehow (e.g., through gametes, the intrauterine environment setting) transmitted across generations. This distinction allows us to organize the main body of epigenetic evidence according to these categories and then focus on the latter (AIE), referring to it as a faster route of informational transmission across generations—compared with genetic inheritance—that guides human evolution in a Lamarckian (i.e., experience-dependent) manner. Of the molecular processes that are implicated in this phenomenon, well-known (methylation) and novel (non-coding RNA, ncRNA) regulatory mechanisms are converging. Our discussion of the chief methods that are used to study epigenetic inheritance highlights the most compelling technical and theoretical problems of this discipline. Experimental suggestions to expand this field are provided, and their practical and ethical implications are discussed extensively.

## Introduction

Many recent studies have demonstrated that stressful conditions that are experienced by parents can influence the offspring’s vulnerability to many pathological conditions, including psychopathologies—primarily related to a disruption in stress response mechanisms. These effects may even endure for several generations. Nevertheless, the mechanisms of this phenomenon have not been detailed, and only recently have scientists examined epigenetics to answer this question.

In this work, we systematize the concept of epigenetic inheritance, discuss the putative mechanisms, and recapitulate the methods for studying this circumstance, presenting their potentialities and limitations. We focus on the transmission of psychopathologies—especially in relation to disruptions in the stress response—because they have long been the center of the historical debate over the weights of genes and the environment in such processes as individual development and inheritance, given their complex nature and clear experience sensitivity. Thus, psychopathologies can be considered one of the most interesting and flourishing fields in the application of epigenetics.

## Historical Background: Filling the Gap

Initially, and for a long time, parental influences on an offspring’s development were focused on two possible sources of variance: genes and the environment. Some scientists concentrated on how “slow and still” information could be transmitted to subsequent generations. The phylogenetic perspective was thus a central assumption, more or less implicit. The premise was that genes themselves carry on blindly: the luckiest genes that are most well suited for the present environmental conditions “win” and endure (Dawkins, 1990).

This idea fits well with the Darwinian concept of adaptation as an all-or-nothing process, which can be recalled easily from the collective imaginary through such terms as “survival,” “reproductive power,” and “law of large numbers.” Those who survive live longer, thus theoretically increasing the probability of finding a mate and reproducing. This can be surely the case, but this theory alone is insufficient to explain phylogenetic development.

Conversely, an alternate perspective has focused on another form of adaptation, residing conceptually inside the lifespan of each individual: ontogenetic development. In the previous gene-centered model, ontogenesis was subordered to phylogenies and was considered a mechanistic unfolding of a predefined genetic program. This idea was progressively revisited when the central “dogma” of molecular biology (proposed by Crick, 1958) was redefined and complemented in light of the growing evidence on the complexity and bidirectionality of gene expression-related processes (Gottlieb, 2007). Soon, the environment appeared through the concept of plasticity and “critical periods” of development. Then, its role became wider when certain authors began discussing “sensitive periods” and environmental programming (see Maccari et al., 2017 for a review).

Yet, for a long time, genes and the environment were considered two separate aspects that interacted at the level of the phenotype. Even epigenetics was conceived of as being able to modify the genetic impact on an individual’s organization but remaining *inside* his existence (i.e., acting only during his lifespan). Until then, there was only one way in which the past could inform the coming new life: genes and parental care. Only when it was demonstrated that epigenetic modifications could be inherited did the ontogenetic (i.e., environmental influences) and phylogenetic (i.e., genetic determinants) worlds—which for years had approached each other in an asymptotic, exhaustive manner—finally merge at a new, theoretical intersection: epigenetic inheritance.

## Epigenetics and Inheritance: Some Definitions

Several handbook definitions should be provided. In general terms, epigenetics is defined as the alterations in the gene expression profile of a cell that are not caused by changes in the DNA sequence (Peschansky and Wahlestedt, 2014). Epigenetic inheritance thus refers to the transmission of certain epigenetic marks to offspring (van Otterdijk and Michels, 2016; Pang et al., 2017). The literature confers different (and sometimes even contrasting) interpretative shades to these terms, underlying distinct aspects of epigenetic modification: their inheritability (e.g., Peschansky and Wahlestedt, 2014; Babenko et al., 2015) environmental sensitivity (e.g., van Otterdijk and Michels, 2016; Bakusic et al., 2017) and stability over time (Houri-Zeevi and Rechavi, 2017).

Epigenetic inheritance is, in certain cases, relegated primarily to paternal contribution (e.g., Rodgers et al., 2013; Gapp et al., 2014; Pang et al., 2017; Yeshurun and Hannan, 2018). Intergenerational epigenetic inheritance represents the transmission of epigenetic marks from one generation to the next—the passage of information from grandparents to a grandchild is instead defined as “transgenerational” (Skinner, 2008; Pang et al., 2017). In fact, many authors (e.g., Babenko et al., 2015; van Otterdijk and Michels, 2016) agree with Skinner’s definition, which allows one to discuss transgenerational epigenetic inheritance only when two criteria are met:

1. exposure to an event in generation F0.
2. an effect of the event must be observed in the third or fourth generation—i.e., F2 or F3—depending on whether the mother or father was first affected (F0).

**Figure 1:**
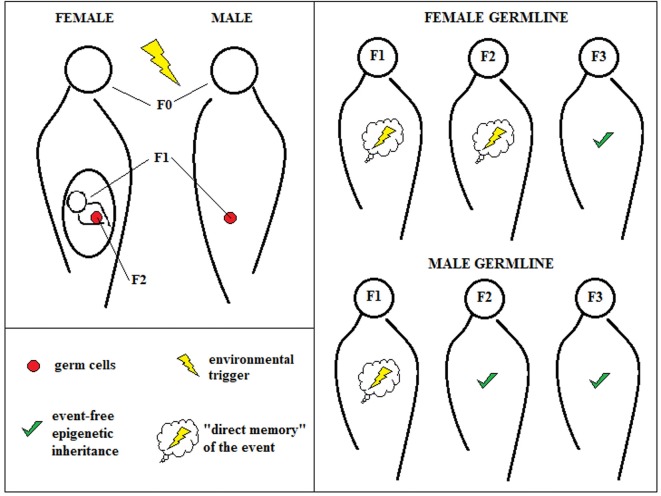
Transgenerational epigenetic inheritance. According to the classical definition of transgenerational epigenetic inheritance, environmental triggers that hit pregnant female individuals (F0) can affect “directly” not only the first new generation (F1), but also its germ cells that represent the second generation (F2). For this reason, only changes in F3 can be due “purely” to epigenetic inheritance. The male germline, instead, can be affected only for one generation, allowing observing epigenetic inheritance already at F2.

Female exposure to a certain environmental factor during pregnancy might even affect the offspring’s germ cells directly, for which reason only the fourth generation can be considered “event-free” and unsullied. When a certain event produces an epigenetic change in the father, it can only modify his sperm, effecting reliable nongenetic inheritance in the third generation (Figure 1).

This definition surely renders the observation of epigenetic inheritance easier, especially in humans, because it prevents the ambiguous interpretation of data that are inevitably contaminated by other events that are not transmitted epigenetically through gamete programming. Nevertheless, this approach excludes the possibility of considering faster epigenetic effects, which are certainly more difficult to control experimentally but could still exist and have functions.

In fact, why must epigenetic transmission occur through germ cells and across several generations? The epigenetic modification of certain genes, produced by an environmental trigger, could lead to significant changes in an individual’s body that could persist over time and in turn signal the epigenetic reorganization of the subsequent generation. This phenomenon could happen without affecting the germline directly and despite the event that fostered such adaptation no being longer active once the embryo has begun its development. As we will see, experimental manipulation in animal models could overcome these problems. For this reason, we will attempt to unify and organize this potentially confusing terminological flowering in a coherent conceptual framework.

## New Conceptualization

In recent years, many scientists have hypothesized and even demonstrated that certain experiences during the life of an individual influence the development of his offspring, even distally. It appears that some experiences modify genetic expression, influencing:

1. how the organism itself responds to a changeable environment (i.e., more ontogenetic flexibility—direct or synchronous effect) and
2. how his descendants will increase their likelihood of surviving in a specific environment—that is, how information is transmitted to offspring regarding the environment that they will encounter (i.e., more phylogenetic flexibility—indirect and both synchronous and asynchronous effects).

### Direct Epigenetics

The first aspect, which we will call direct epigenetics (DE), comprises all of the epigenetic changes that occur during an individual’s lifespan. Notably, this phenomenon implies even dynamic and short-term regulation of gene expression, mediated by the action—almost in real time—of regulatory proteins, called transcription factors, such as c-fos, c-jun, ZENK and CREB. The genes that encode for such crucial functional elements are called immediate-early genes, because a change in their expression is the first event that launches cascades of adaptive events, including the transcriptional aspects of other genes (Johnson, 2010), ultimately producing even long-lasting effects.

As we will discuss, these factors regulate the expression of genes that encode for other functional proteins, as well as for different classes of regulatory elements, such as non-coding RNAs (ncRNAs), that can mediate epigenetic processes. Recently, some authors have highlighted the function of ncRNAs in regulating, more or less directly, several epigenetic processes in the development of an individual and in epigenetic inheritance (Peschansky and Wahlestedt, 2014; Bohacek and Mansuy, 2015; van Otterdijk and Michels, 2016). However, the debate over whether ncRNAs should be considered epigenetic factors continues (Kovalchuk, 2012). We propose, albeit cautiously, to follow the literature that considers them as such (e.g., Babenko et al., 2015), because they appear to meet our wide definition of epigenetics.

Epigenetic mechanisms of DE are potentially countless. Here, we have mentioned transcription factors as an extreme example, because they highlight what is the main attribute of DE: its high contingency. Below, we will focus on methylation and ncRNA action, because they are so far considered the most likely linking mechanisms between ontogenetic and phylogenetic development. Indeed, all of the possible transient changes to gene expression that we have described can have irreversible effects in the long term that can even become part of the epigenetic burden that is transmitted to the offspring (point 2, above). This appears to be particularly true for the first several months of life, and as we discuss, many epigenetic studies have focused on sensitive periods (Jawahar et al., 2015; Maccari et al., 2017). The importance of experiences in the first period of life in establishing the development of an individual has long been acknowledged, even before the discovery of the human genome (consider Freud’s and Bowlby’s insightful works), but only recently have these old theories been reintroduced in light of the latest findings on epigenetic mechanisms. Among them, we consider Fagioli’s Human Birth Theory that posits that mental illness develops primarily during the postnatal period (approximately within the first year of life) but becomes clinically evident later in life (Maccari et al., 2017). Although this type of theory is favored by a significant amount of evidence, as we have argued before, they are likely just a component (even though an extremely important one).

### Indirect Epigenetics

When an epigenetic change produced by a direct experience (DE) is transmitted to the offspring, that same experience becomes an indirect environmental trigger for the ontogenetic development of the new individual. Paralleling Crews (2008) and van Otterdijk and Michels (2016), the second form of environmental action (i.e., phylogenetic adaptation) can be divided into two categories of “indirect epigenetics (IE):” within and across. These two aspects can be considered the conceptual product of the historical development of this matter, the latter (across) being a more recent acquisition. Theoretically, these components are related and difficult to distinguish, even operationally.

Within indirect epigenetics (WIE) encompasses all of the epigenetic changes that act synchronously on the developing individual. Temporally, it starts at the very moment at which the zygote is formed and the environment begins changing. This category includes all of the factors that, more or less indirectly, can affect the developing individual, from the start to end of gestation. The underlying concept is that environmental changes occur when the (proto)-individual actually exists, synchronously.

Across indirect epigenetics (AIE) describes what happens from the moment of conception back toward the parents’ earlier life experiences (and even grandparents, as we will discuss), which asynchronously set the composition of germ cells (and possibly that of the intrauterine environment). Some authors have referred to all epigenetic changes that appear to be transmitted across generations as epimutations, in contrast to classical, less frequent genetic mutations (Bennett-Baker et al., 2003). Notably, in this case, a certain event has consequences that are maintained over time, affecting the offspring’s destiny during gestation and, most importantly, later in life. Clearly, it is reasonable to believe that the closer we are to the moment of conception, the stronger the prediction power of the variable is, or at least the easier it is to hypothesize a “causal” relationship, because it should be expected in an epistemology of complexity that conceives of development in terms of probabilistic epigenesis (see Gottlieb, 2007 for a theoretical detailed explanation). Nevertheless, as we will see, certain events can act as relevant predictors even when distal in time.

We can discuss epigenetics only if a modification to gene expression takes place. This idea, supported by Kovalchuk (2012), renders the function of the intrauterine environment in epigenetic transmission controversial—in cases in which environmental events produce changes that do not affect germ cells directly but persist and affect the newborn in later gestational stages. Nevertheless—and for this same reason—the epigenetic mechanisms that determine the womb cannot be neglected if they are demonstrated to mediate the transmission of information on the genetic expression of the developing organism (as discussed below). As we will see, of all of the epigenetic mechanisms that are implied in these two indirect forms of transmission, maintenance methylation, *de novo* methylation and the regulatory and amplifying activities of ncRNA, are the most prominent. Although increasing data strongly suggest transgenerational inheritance of epigenetic information, the non-DNA-based processes by which information is transmitted across generations are largely unknown (Houri-Zeevi and Rechavi, 2017).

### Wider Clarifications and Considerations

The categorization above represents a mere conceptual distinction that has been conceived simply to elucidate the phenomenon of interest. As a matter of fact, all of these aspects are expected to interact continuously, but we can distinguish, on a case-by-case basis, which conceptual element (ontogenetic vs. phylogenetic, direct vs. indirect, or even within vs. between) has more apparent relevance. Notably, our aim is to indicate that relegating epigenetic transmission only to the moment of gestation is imprecise, hindering us from developing a wider and exhaustive understanding of this phenomenon. Moreover, our purpose is to merge and soften all dichotomic types of conceptualization, including those that we have proposed herein.

Wider environmental effectors, such as parental style and cultural aspects, must be considered with caution. They seem to be direct and indirect in their action, as well as synchronous and asynchronous. Certainly, they account for the general setting in which the newborn develops, which in turn begets different and complex forms of information about the past that guides ontogenetic and phylogenetic adaptation. This should be considered the most intuitive means of transgenerational transmission of information—the most naïve but still undeniable Lamarckian addition to Darwinian evolution. Nevertheless, and for this reason, the effect of these two variables is too complicated to account for, and studies that have attempted to demonstrate their function in epigenetic transmission (as we will see in the next section) are not exhaustive. Moreover, a discussion of these wider environmental factors is not pertinent to our discourse, given the level of inquiry that we are considering. Thus, we are setting aside these two aspects from our argumentation, except for prenatal maternal care and the few historical events that have been suggestive objects of study (e.g., the Dutch Famine and the Holocaust).

Epigenetic changes that can be transmitted can belong to any of the previous categories (DE and IE). In the strict concept of transgenerational epigenetic inheritance, the “marking” event can happen before or during conception (Gapp et al., 2014), but certain effects can legitimately be considered to be epigenetically inherited after a certain number of generations and in the absence of the same environmental event (Skinner, 2008). Adopting a wider and more complex perspective, the additional epigenetic role of external epigenetic cues (i.e., molecular signals from parents that influence the epigenetic setting of the zygote) should be taken into greater account. Moreover, the idea that an environmental factor should not be repeated in the lifespan of the offspring is experimentally clever but still forms a chasm between science and the reality that it is supposed to inquire: the central concept of epigenetic inheritance is that information about the environment is passed to the next generation. These mechanisms can lead to errors and anomalies, but we should at least consider and test what happens when the “predicted” event occurs (Godfrey et al., 2007).

### Epigenetic Spacetime

What does the concept of epigenetic inheritance add to science with respect to the earlier concept of evolution and genetic transmission? This type of communication appears to be faster and more contingent and thus more efficient. For this reason, epigenetics increases our heuristic power through a different concept of evolution, in which the environment has a more proactive role in influencing communication across generations, depending ultimately on two interconnected evolutionary processes: a Darwinian process (slow but steady) and a Lamarckian process (quick but labile). The historical contraposition of these processes is now evolving into a unified theory of evolution (Skinner, 2015).

We propose this informational unfolding to happen in spacetime that is conceived of as a four-dimensional field, in which events are vectors that assume their coordinates (s1, s2, s3, t) depending on the observation point. Borrowing this definition from physics, however, is not sufficient for describing ontogenetic and phylogenetic processes, because it neglects the cyclical nature of spacetime occurrences, which is crucial for understanding natural processes on the biological scale, such as epigenetics (Masri and Sassone-Corsi, 2013; Stevenson and Prendergast, 2013; Azzi et al., 2014). When examining local processes, space and time can be addressed artificially as distinct and independent: in this case, time can be intended as a directional flow that is governed by biological rhythms. On medium and large scales, combining these two perspectives, we propose a model in which spacetime evolves through a complex, coil-like pathway in a nonlinear—but stochastically defined—direction, constantly producing different, multilevel open cycles. These revolutions in 4D space can overlap and appear to be identical only when observed from a certain perspective, creating an illusion of circularity (e.g., circadian rhythms, the succession of generations).

**Figure 2:**
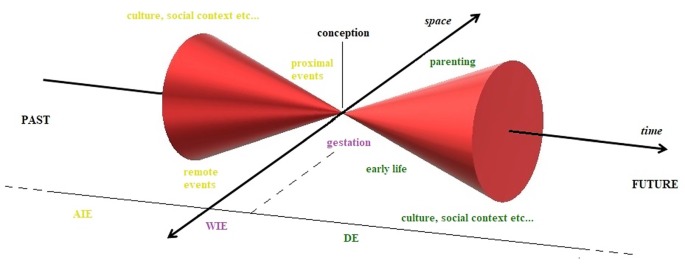
Epigenetics through the Minkowskian cone. Epigenetic changes and related environmental factors visualized in 4D Minkowskian space, assuming conception as our arbitrarily chosen observation point, the zero of the system. Across indirect epigenetics (AIE) includes all those adaptations in parental life that precede conception; within indirect epigenetics (WIE) describes all those changes that take place during the gestational period and, finally, direct epigenetics (DE) describes all those plastic processes that can occur after birth. Although these processes are strongly interconnected and can overlap on multiple levels in a complex real system, here they are treated as discrete and sequential, for the sake of clarity.

Therefore, we can imagine the transmission of information across generations as a succession of cycles, a sequence of light-cones that represent the multidimensionality of a theoretical wave function that describes the amplitude of indetermination or the potential of the evolving system. Thus, we can picture the “pulsing” of this probabilistic informational mass unfolding across spacetime, merging at the moment of conception (considered our arbitrarily chosen observation point) and then expanding, only to collapse again (Figure 2).

## Evidence of Epigenetic Changes

There are several reviews on epigenetic changes (Jawahar et al., 2015; Jung and Pfeifer, 2015; Conti and Alvares da Silva-Conforti, 2016; Maccari et al., 2017) and their heritability across generations (Gapp et al., 2014; Skinner, 2014; Babenko et al., 2015; Bohacek and Mansuy, 2015; Szyf, 2015; van Otterdijk and Michels, 2016; Ambeskovic et al., 2017; Pang et al., 2017; Yeshurun and Hannan, 2018). We report only some of the most significant evidence to provide concrete examples of our proposed classification.

### Evidence of Direct Epigenetics

Epigenetic changes can be triggered by several environmental factors, such as diet (Mathers et al., 2010), pollution (Christensen and Marsit, 2011), smoking (Talikka et al., 2012), that can be labeled generically as “stressors,” referring to the neutral, adaptive meaning of the term (Cabib and Puglisi-Allegra, 2012). Epigenetic aberrations have been implicated in many diseases, primarily cancer but also cardiovascular, autoimmune, metabolic and neurodegenerative diseases, often with particular regard to aging (van Otterdijk et al., 2013; Jung and Pfeifer, 2015).

Many groups have demonstrated the function of epigenetic mechanisms in mediating the risk and development of certain psychopathologies (Faa et al., 2016; O’Connor et al., 2016; Maccari et al., 2017)—particularly anxiety and depression (e.g., Szyf, 2015; Andolina et al., 2016). For instance, adverse early experiences (e.g., low maternal care, abuse) can affect glucocorticoid receptor (GR) gene (NR3C1) expression, which stably predisposes one to anxiety and depression (Smart et al., 2015; Conti and Alvares da Silva-Conforti, 2016). As discussed, there is increasing evidence for ncRNAs regulating several adaptive processes in the life-span, which, according to our definition, can also be considered a form of DE.

The expression patterns of microRNAs (miRNAs), for example, have been studied in correlation with neuro- and psychopathologies, such as Alzheimer’s disease, Parkinson’s disease, Huntington’s disease, multiple sclerosis, schizophrenia, addiction, autism, bipolar disorder and especially anxiety and depression (Baltimore et al., 2015; O’Connor et al., 2016). Stress is the most extensively studied trigger of alterations to miRNAs, primarily in the brain (Hollins and Cairns, 2016), and certain expression patterns of this subclass of short ncRNAs have been associated with several measures of anxiety and depression—most notably stress-dependent psychopathologies.

#### Animal Models

Maternal care behavior, the first environment for the newborn, effects several epigenetic changes in the offspring. Quality of maternal care appears to predict alterations in DNA methylation in the offspring. In animal models, maternal care behaviors, such has licking, grooming, and arched-back nursing, can alter DNA methylation patterns and chromatin structure, particularly in the gene that encodes the GR (Francis et al., 1999; Weaver et al., 2004). Maternal separation, considered the archetype of early life stress, is associated with alterations in methylation patterns in several genes that are implicated in anxiety (Murgatroyd et al., 2009; Kember et al., 2012; Wu et al., 2014)—notably corticotropin-releasing factor (CRF) and its receptor (Chen et al., 2012; Sotnikov et al., 2014).

Several studies have examined the regulatory effects of adult stress on the methylation of the NR3C1 gene as a pathological marker and mediator of pathology, consequent to dysregulation of the hypothalamic–pituitary–adrenal axis (HPA), as exemplified in animal models of social defeat stress (St-Cyr and McGowan, 2015). In addition, brain-derived neurotrophic factor (BDNF) is downregulated in several areas of the brain in animal models of depression (Elfving et al., 2010; Molteni et al., 2010; Qiao et al., 2014). Methylation of the promoter region of *BDNF* is associated with a reduction in hippocampal volume. In several animal models, hippocampal BDNF levels decline under acute (Barrientos et al., 2003) and chronic (Nibuya et al., 1995) stress conditions. Antidepressant treatment upregulates hippocampal BDNF, and knocking out *BDNF* in animal models impairs the response to treatment (Khundakar and Zetterström, 2006; Monteggia et al., 2007).

Illustrating the unique functions of immediate-early genes (IEGs; cited above), CREB-mediated transient plasticity mediates the transition to an irreversible phenotype of addiction through the accumulation of delta-FosB, which mediates structural synaptic readaptations that strengthen themselves in a positive-feedback process (Koob and Volkow, 2010).

As reviewed by Kolshus et al. (2014), there is much evidence of altered miRNA expression in depression and greater expression after antidepressant treatment (e.g., miR-16, miR-30, miR-128, miR-132, miR335, miR-494, miR182). For example, miR-16 levels are higher in raphe nuclei after administration of fluoxetine, decreasing serotonin transporter levels and thus enhancing serotoninergic transmission, ultimately reducing depression-like behaviors (Baudry et al., 2010). Moreover, miR-16 is downregulated in the locus coeruleus and the hippocampus (Launay et al., 2011). Considering the hypothesis of the regulation of HPA and related anxiety-like behaviors by miRNAs, miRNA levels in the prefrontal cortex and the amygdala have been studied with regard to fear extinction and maternal separation (Kolshus et al., 2014). Notably, in a model of learned helplessness, rats that exhibit learned helplessness (i.e., freezing instead of avoiding a previously inescapable stimulus) have lower miRNA levels, relative to other animals that continue to try to escape (Smalheiser et al., 2011).

Cohen et al. (2017) have reported disparate patterns of miRNA expression in the amygdala and dorsal raphe nuclei (DRN) in two groups of rats, divided into high and low responders after exposure to chronic stress, depending on the coping strategy that is adopted (active and passive, respectively). MiR-34c is upregulated in the central amygdala after acute and chronic stress and represses several stress-related proteins, such as CRFR1, thus mediating anxiolytic effects (Haramati et al., 2011). Andolina et al. (2016) recently demonstrated that miR-34 mediates anxiety-like behaviors and fear responses, wherein its absence in a knockout (KO) model engenders a stress-resilient phenotype and favors fear extinction. Particularly, wild-type (WT) mice overexpressed miR-34c in the basolateral amygdala (BLA) but not the medial prefrontal cortex (mPFC), whereas miR-34a levels increased only in the mPFC. These changes were accompanied by neurochemical, morphological and behavioral alterations. The absence of miR-34 mitigated stress responsivity and facilitated fear extinction.

The levels of certain stress-related proteins have been analyzed. CRFR1 levels are elevated in total KO (TKO) mice, as reported by Haramati et al. (2011). Similarly, Andolina et al. (2018) examined the effects of the absence of miR-34a/b/c (i.e., a TKO model, or TKO) on coping behavior. MiR-34 levels were assessed in all of the main areas that are involved in stress responses, peaking in DRN. TKO mice tended to resort to active coping behaviors when challenged by the forced swim test (FST). Accordingly, TKO mice overexpressed CRFR1 in DRN, compared with WT mice, and their “resilient” behavioral phenotype could be reverted through the injection of a CRFR1 antagonist, confirming its function in regulating stress response strategies.

miR-34 TKO mice lack the stress-induced regulatory neurotransmission (5-HT mPFC/GABA BLA release) that contributes to passive coping behavior (Andolina et al., 2013, 2014). Recent results (Andolina et al., 2018) highlight the molecular mechanisms of this phenomenon, completing this model: when a stressor is encountered, miR-34 levels influence which coping strategy will be implemented through its inhibition of CRFR1 expression in DRN, which in turn regulates the balance in cortico-subcortical 5-HT/GABA activity that ultimately governs motivation and its behavioral output (Puglisi-Allegra and Andolina, 2015).

#### Clinical Evidence

There is evidence of epigenetic changes in humans throughout life and several extensive reviews, many of which have focused on alterations in the stress response and psychopathologies (Faa et al., 2016; O’Connor et al., 2016; Maccari et al., 2017). Anxious individuals have higher global methylation levels compared with nonanxious controls (Murphy et al., 2015). Specifically, social anxiety disorder, for example, is associated with decreased OXTR methylation and greater cortisol release and amygdalar activation in response to anxiety-related triggers (Ziegler et al., 2015). Moreover, monoamine oxidase A (MAOA) methylation levels are lower in patients with panic disorder vs. healthy controls, and only patients who respond to cognitive-behavioral therapy experience upregulation of MAOA (Ziegler et al., 2016). Another important gene, glutamate-decarboxylase 1 (GAD1), encoding a crucial glutamatergic metabolic factor, is undermethylated in patients with panic disorder (Domschke et al., 2013). Chronic psychosocial stress has been associated with hypermethylation of the NR3C1 gene (Witzmann et al., 2012).

Several studies have reported conflicting results on the methylation levels of these genes and hyper/hypocortisolism in depressed patients (Bakusic et al., 2017). For example, HPA axis hyporeactivity has been observed in depressed patients, associated with overexpression (OE) of GRs (Vangeel et al., 2015). In contrast, a previous study has shown a reduction in GR-stimulated gene expression with higher blood levels of cortisol in major depressive disorder (Menke et al., 2012). Notably, antidepressant treatment alters BDNF expression in the prefrontal cortex in humans (Chen et al., 2011). Finally, higher methylation levels in the serotonin transporter gene (SLC6A4) correlates with a positive response to psychotherapy (Roberts et al., 2014).

The function of various miRNA families in environmental adaptation has been established in clinical studies. Altered peripheral levels of several miRNAs have been seen in many psychiatric disorders, such as bipolar disorder, major depressive disorder, anxiety disorders and schizophrenia (O’Connor et al., 2016). The causal relationships with such epigenetic markers, however, are often undefined. For example, the blood levels of miR-663 correlate negatively with psychiatric symptoms of anxiety (Chen S. et al., 2016). Similarly, by microarray, the expression of several miRNAs in the blood of bipolar and depressed patients has been measured, wherein disorder-specific and commonly altered miRNAs have been identified (Maffioletti et al., 2016).

In these cases, such alterations in epigenetic markers could be caused by genetic polymorphisms, epigenetic adaptation to certain environmental conditions, or both. MiR-135 is downregulated in depressed patients and has been proposed to be an “endogenous” antidepressant, based on its sensitivity to selective serotonin reuptake inhibitors (SSRIs; Issler et al., 2014). In patients with major depressive disorder who respond poorly to antidepressant treatment, the blood levels of several miRNAs are altered, particularly those that are involved in nucleotide binding and chromatin assembly; consequently, four putative miRNAs that were predictive of treatment outcome were identified (Belzeaux et al., 2012). Given the technical constraints of clinical research, there is little direct evidence of altered patterns in the brain. Several postmortem studies have reported significant downregulation of key miRNAs in the prefrontal cortex of depressed suicide subjects (Smalheiser et al., 2012).

### Evidence of Within Indirect Epigenetics

There are many factors that affect the offspring epigenetically during pregnancy, in a process that is termed fetal programming (Faa et al., 2016; Maccari et al., 2017). The concept of fetal programming refers to all of the adaptations to the intrauterine and maternal environment that shape the developing individual structurally and functionally (Swanson et al., 2009). These processes can affect, in particular, brain development, possibly leading to neuropsychiatric disorders, such as Parkinson’s disease, Alzheimer’s disease, attention-deficit hyperactivity disorder, schizophrenia, bipolar disorder, major depressive disorder and anxiety disorders (Faa et al., 2016).

Maternal epigenetic factors that are active during gestation and interfere with neurodevelopment have been grouped into two overarching categories: maternal and fetal. Maternal factors include maternal diet, smoking, alcoholism, hypertension, malnutrition, trace elements, stress, diabetes, substance use and exposure to environmental toxicants (Al-Gubory, 2014; Lyall et al., 2014). Maternal hormones, immune factors, nutrients and odors can be even modified by the presence of the father, who thus appears to have an influence even during gestation (Todrank et al., 2011). Fetal factors consist of the hazards that cause intrauterine restriction, such as fetal hypoxia/asphyxia, placental insufficiency, prematurity, low birth weight and drugs that are administered to the mother or baby in the perinatal period (Hunter et al., 2016).

#### Animal Models

Animal models have been used to demonstrate the indirect effects of environmental factors during pregnancy on the offspring’s development. For example, maternal fat diet increases the susceptibility of male offspring to liver disease through epigenetic reprogramming of lipid metabolism and inflammatory responses (Pruis et al., 2014). Further, prenatal undernutrition, for example, can permanently alter DNA methylation in the sperm of adult offspring in regions that are resistant to zygotic reprogramming, potentiating transgenerational transmission of metabolic disorders (Radford et al., 2014).

Maternal immune activation, in contrast, predisposes the offspring to depression (Ronovsky et al., 2017), and certain odors influence olfactory neurodevelopment and shape preferences for certain scents (Todrank et al., 2011)—in particular, the odor of a predator affects the stress response epigenetically in offspring (St-Cyr and McGowan, 2015). Exposure to testosterone during pregnancy—for example, due to polycystic ovary syndrome—can affect the limbic system of offspring in rats and contribute to elevated anxiety-like behavior (Hu et al., 2015). Experience with prenatal gastrointestinal stress in rodent dams engenders anxiety-like and depression-like behaviors in adult offspring (Zheng et al., 2016).

Fetal programming also depends on miRNAs, although there is limited evidence in WIE. Placental miRNAs have been implicated by several groups (Maccani et al., 2013; Morales-Prieto et al., 2014), but there is still scarce proof of their actual involvement and there is no direct evidence of the epigenetic changes that consequently occur in the developing fetus.

#### Clinical Evidence

There are many studies on the effects of the maternal environment during pregnancy on offspring in humans, but none demonstrated that these outcomes are mediated by epigenetics directly. As reviewed by Faa et al. (2016), a lack of protein, iron, choline, or zinc can precipitate several cognitive deficits and even intellectual disabilities and autistic symptoms in the offspring. Similarly, smoking during pregnancy favors premature birth and motor, memory and behavioral deficits. Stress is a significant mediator of vulnerability, and it has been hypothesized that excess cortisol, due to maternal anxiety, induces neurodevelopmental damage in the fetus after crossing the placental barrier (Dorrington et al., 2014). Maternal stress can disrupt GABAergic inhibitory transmission, leading to anxiety or maladaptation to stressors in the offspring (Fine et al., 2014).

Conradt et al. (2013) reported increased placental 11-beta hydroxysteroid dehydrogenase type 2 (11β-HSD2) methylation following fetal exposure to maternal anxiety and greater placental NR3C1 methylation when the mother was depressed during pregnancy; in both cases, the offspring was in a heightened hypotonic state. 11β-HSD2 is an enzyme that has been suggested to regulate placental permeability to the mother’s circulating hormones, such as cortisol. Impaired production of this protein results in a leaky placenta that does not adequately protect the offspring from the detrimental effects of excess cortisol. Notably, licorice consumption during pregnancy enhances the permeability of the placenta to glucocorticoids through methylation of the 11β-HSD2 gene, altering the HPA response in the offspring (Räikkönen et al., 2011). Depressed and anxious mothers, regardless of treatment with SSRIs during pregnancy, have higher levels of placental 11β-HSD2, SLC6A4 (serotonin transporter), and SLC6A2 (norepinephrine transporter) compared with healthy controls (Ponder et al., 2011).

There is even less evidence on the function of miRNAs in epigenetic changes during fetal development. Maternal smoking during pregnancy is associated with the downregulation of miR-16, miR-21, and miR-146a in the placenta (Maccani et al., 2010). miR-146 is altered following the exposure of immortalized trophoblastic cells to bisphenol A, a synthetic organic compound that is used in the manufacture of epoxy resins and other polymers (Avissar-Whiting et al., 2010). MiR-16 and miR-146 have thus been implicated as responsive mechanisms to cell stress (Morales-Prieto et al., 2014).

### Evidence of Across Indirect Epigenetics and Transgenerational Epigenetic Inheritance

Across indirect epigenetic changes, *per se*, define only intergenerational epigenetic inheritance, which is inheritance from one generation to the next (Pang et al., 2017). AIE can be and has been considered, instead, a necessary but insufficient condition for transgenerational epigenetic inheritance, at least per its canonical definition (Skinner, 2008). Many experiments have been performed to prove some form of epigenetic inheritance in the past two decades. We report several examples below.

#### Animal Models

Rat malnutrition impairs cognition in offspring (Galler and Seelig, 1981). A low-protein diet over 10 generations produces even more severe cognitive deficits, which are evident after two generations, on returning to a regular diet (Stewart et al., 1980). Dunn and Bale (2009) have demonstrated that a maternal high-fat diet in mice increases body size and insulin sensitivity, which endure until the second generation; these effects nearly vanish in the F3 generation, despite the alterations in body size being observed solely in female offspring, suggesting an imprinting mechanism. Parental addiction in rodents alter the sensitivity of offspring to drugs, eliciting adaptive counterregulatory responses (Byrnes et al., 2011; Vassoler et al., 2013; Finegersh and Homanics, 2014).

Environmental exposure to vinclozolin, an endocrine disruptor that is commonly used as an agricultural fungicide, increases sensitivity to stress—namely, anxious behavior—in the F3 generation (Crews et al., 2012). At the molecular level, several abnormalities have been observed, such as DNA methylation in the male testis of F1 animals, which impairs spermatogenic capacity (Anway et al., 2005); altered methylation of several imprinting sites in F1 (Stouder and Paoloni-Giacobino, 2010); altered metabolic brain activity, testosterone levels in response to stress, and hippocampal gene expression (Crews et al., 2012); changes in germ cells (Skinner et al., 2013); and diseases of the reproductive organs (Manikkam et al., 2012). Fetal exposure to alcohol or vinclozolin heightens the sensitivity of newborn rats and their two ensuing generations to stress (Govorko et al., 2012).

Chronic, unpredictable traumatic experiences in early postnatal life alter social recognition, and chronic social instability in adolescence disrupts social interactions across three generations; these properties are transmitted through the germ cells of male and female mice, despite the former failing to express any symptoms (Franklin et al., 2011; Saavedra-Rodríguez and Feig, 2013). Postnatal trauma elicits depressive-like behaviors that are evident for up to three generations, even after crossfostering (Franklin et al., 2010). These changes are associated with altered DNA methylation levels in the brain and sperm and have been interpreted as a change in the stress response. Repeated social stress in male mice during adolescence increases behavioral despair and anxiety in their offspring (Dietz et al., 2011).

In a study by Yao et al. (2014), stressing pregnant dams (F0) enhanced the risk of a shortened gestation for up to the third generation; moreover, when the stress was present in the subsequent generation, F3 offspring developed sensorimotor impairments. These abnormalities were associated with the upregulation of miR-200b and downregulation of miR-429. Restraining male and female mice for 60 days increased the mRNA levels of GR and BDNF and mitigated anxious-like behaviors in F1 and F2 offspring (He et al., 2016). Paternal chronic stress sensitizes F1 animals to stress and evokes depressive-like behaviors, in association with altered miRNA expression in sperm in F2 (Morgan and Bale, 2011).

A similar stress paradigm was applied to demonstrate that paternal stress alters sperm miRNA levels, perhaps mediating the disruptions in stress response in subsequent generations (Rodgers et al., 2013). To verify this function of mRNA, the nine overexpressed mRNAs reported by (Rodgers et al. (2013); miR-193–5p, miR-204, miR-29c, miR-30a, miR-30c, miR-32, miR-375, miR-532–3p and miR-698) were injected into a zygote, and similar glucocorticoid levels and behavioral responses as in the offspring of stressed male mice were observed in the offspring (Rodgers et al., 2015). The authors suggested that miRNA expression in sperm silences maternal gene expression and epigenetically alters the developmental fate of subsequent generations.

In Short et al. (2016), the administration of corticosterone to male mice for 2 months before mating affected fear and anxiety responses in the F1 generation in a sex-dependent manner. Further, the paternally imprinted gene Igf2 was overexpressed and underexpressed in the hippocampus of males and females, respectively. F2 offspring exhibited lower levels of anxiety, but only males developed a depressive-like phenotype. The levels of miR-98, miR-144 and miR-190b were altered in the sperm of F0 males, and thus, they were regarded as putative mediators of the epigenetic effects of corticosterone across generations. Notably, environmental enrichment reverted some of the adverse outcomes of the stress that was experienced by grandparents (Leshem and Schulkin, 2012) and improved memory in subsequent generations (Arai et al., 2009).

In addition to the effects of negative environments across generations, recent studies have begun to examine those of positive conditions. Enhanced cognitive stimulation and physical activity reduce the response to adult stress, but only recently have the transgenerational effects of enrichment of the paternal environment on the offspring been evaluated. Anxiety-like and depression-like behaviors and biomarkers of the stress response have been assessed in F1 and F2 descendants from male mice that have been exposed to environmental enrichment (F0). A sex-dependent effect on stress responsivity emerged in the F2 generation (Yeshurun et al., 2017). Short et al. (2017) showed that paternal exercise significantly alters the small ncRNA content of sperm—an effect that was associated with an anxiolytic behavioral phenotype in male offspring. In particular, three miRNA classes (miR-19b, miR-455 and miR-133a) and two species of transfer-derived RNAs (tRNA-Gly and tRNA-Pro) were modified in sperm (Short et al., 2017).

#### Clinical Evidence

Direct proof of transgenerational epigenetic inheritance in humans remains lacking (van Otterdijk and Michels, 2016). Nevertheless, there is notable indirect evidence (i.e., longitudinal studies with no or few insights into putative epigenetic mechanisms).

Male children who were exposed to intrauterine undernourishment during the 5-month Dutch famine (occurring in 1944–1945) and their offspring developed obesity, glucose intolerance and coronary heart disease in adult life (Painter et al., 2008; Lumey et al., 2011; Veenendaal et al., 2013). In some cases, these symptoms were associated with altered levels of DNA methylation 60 years later (Heijmans et al., 2008). Further, the risk of diseases is higher when gestational famine is followed by a calorie-rich diet later in life (Schulz, 2010). The Överkalix cohort study has reported the effects of ample or poor food availability to Norwegian children and adolescents on the longevity of their descendants showing a risk of death due to diabetes (Kaati et al., 2002) and increased lifespan in grandchildren, respectively (Bygren et al., 2001).

Transgenerational transmission of trauma has been studied in the offspring of Holocaust survivors, combat veterans, and refugee families (Vaage et al., 2011; Kellermann, 2013). A Norwegian longitudinal study on Vietnamese refugees reported a high risk of mental disease in F3 offspring, when grandparents were diagnosed with post-traumatic stress disorder on their arrival in Norway (Vaage et al., 2011).

### Summary

There is copious evidence of epigenetic changes in animal models, but this field must improve to generate stronger evidence and implement new techniques that could apply to human studies, in which direct and robust proof remains lacking. We have compiled many studies and divided them by epigenetic type and research model (Table 1). As discussed, this review’s aim is not to report all existing studies in the field but to provide some examples that can help us better understand epigenetic changes and their inheritance.

**Table 1:** Evidences of the three defined forms of epigenetic changes: direct epigenetics (DE), within indirect epigenetics (WIE) and across indirect epigenetics (AIE).

	Animal model	Human model
**DE**	Nibuya et al. (1995)	Molteni et al. (2010)
Changes that occur within the lifespan of an individual, due to the direct experience of his/her environment.	Francis et al. (1999)	Chen et al. (2011)
	Barrientos et al. (2003)	Belzeaux et al. (2012)
	Weaver et al. (2004)	Menke et al. (2012)
	Karege et al. (2005)	Smalheiser et al. (2012)
	Khundakar and Zetterström (2006)	Vangeel et al. (2015)
	Monteggia et al. (2007)	Witzmann et al. (2012)
	Baudry et al. (2010)	Domschke et al. (2013)
	Elfving et al. (2010)	Issler et al. (2014)
	Molteni et al. (2010)	Murphy et al. (2015)
	Murgatroyd et al. (2009)	Ziegler et al. (2015)
	Haramati et al. (2011)	Chen S. et al. (2016)
	Launay et al. (2011)	O’Connor et al. (2016)
	Smalheiser et al. (2011)	Maffioletti et al. (2016)
	Chen et al. (2012)	Ziegler et al. (2016)
	Kember et al. (2012)	Bakusic et al. (2017)
	Qiao et al. (2014)	
	Sotnikov et al. (2014)	
	Wu et al. (2014)	
	St-Cyr and McGowan (2015)	
	Andolina et al. (2016, 2018)	
	Cohen et al. (2017)	
**WIE**	Todrank et al. (2011)	Avissar-Whiting et al. (2010)
Changes that occur inside the womb, due to events that take place during gestation.	Pruis et al. (2014)	Maccani et al. (2010)
	Radford et al. (2014)	Ponder et al. (2011)
	Hu et al. (2015)	Räikkönen et al. (2011)
	St-Cyr and McGowan (2015)	Conradt et al. (2013)
	Zheng et al. (2016)	Dorrington et al. (2014)
	Ronovsky et al. (2017)	Fine et al. (2014)
**AIE**	Stewart et al. (1980)	Bygren et al. (2001)
Changes that affect an individual’s predecessors (parents, grandparents etc.), due to events that occur even long before conception and that are somehow transmitted across generation.	Galler and Seelig (1981)	Kaati et al. (2002)
	Arai et al. (2009)	Heijmans et al. (2008)
	Dunn and Bale (2009)	Painter et al. (2008)
	Anway et al. (2005)	Schulz (2010)
	Franklin et al. (2010)	Lumey et al. (2011)
	Stouder and Paoloni-Giacobino (2010)	Vaage et al. (2011)
	Byrnes et al. (2011)	Kellermann (2013)
	Dietz et al. (2011)	Veenendaal et al. (2013)
	Franklin et al. (2011)	
	Morgan and Bale (2011)	
	Crews et al. (2012)	
	Govorko et al. (2012)	
	Leshem and Schulkin (2012)	
	Manikkam et al. (2012)	
	Rodgers et al. (2013)	
	Saavedra-Rodríguez and Feig (2013)
	Skinner et al. (2013)	
	Vassoler et al. (2013)	
	Finegersh and Homanics (2014)
	Yao et al. (2014)	
	Rodgers et al. (2015)	
	He et al. (2016)	
	Short et al. (2016)	
	Short et al. (2017)	
	Yeshurun et al. (2017)	

*The definition of the categories is also provided*.

## Epigenetic Mechanisms

How does epigenetic inheritance occur concretely? Although several epigenetic processes have been considered to answer this question, given the wide range of this work, we will focus on two of the more extensively studied mechanisms: methylation and ncRNA.

### First-Generation Epigenetic Mechanisms

First-generation epigenetic mechanisms are centered on modifications to chromatin density—i.e., a “tuning” of transcriptional probability. These mechanisms depend on several enzymatic activities that effect acetylation, methylation and phosphorylation of histone tails (primarily lysine, arginine and serine) and their removal (deacetylation, demethylation and dephosphorylation); ATP-dependent chromatin remodeling (proteins that actively and transiently modify nucleosomal structure); and cytosine methylation (Portela and Esteller, 2010; Cooper and Hausman, 2013). Although these processes might mediate epigenetic inheritance, methylation is the most well-understood mechanism regarding this matter (Babenko et al., 2015).

#### Methylation and Demethylation

DNA methylation is an enzymatic process by which a methyl group (CH_3_) is covalently bound to the fifth position of a cytosine residue (5-methylcytosine, 5mC) to alter gene expression. In mammalian DNA, this regulatory activity acts on CpG palindromes (i.e., diagonally symmetric couples of guanine-cytosine pairs), whereas asymmetric methylation is rare (Chen and Li, 2004). When methylation affects the promoter region, it is associated with gene silencing—the most well-known function of this mechanism; however, when it involves the transcribed region, it increases transcriptional activity (Jones, 2012). DNA methylation is involved in many processes, particularly those that are important for early development, such as genomic imprinting, X-chromosome inactivation, and transposon silencing (Smith and Meissner, 2013).

The addition of CH_3_ groups to CpG islands is catalyzed by DNA methyltransferases (DNMTs). DNMT1 primarily maintains DNA methylation patterns during replication, whereas DNMT3A, DNMT3B and DNMT3L (a noncatalytic isoform of DNMT3, termed DNMT3-like) are principally involved in establishing new DNA methylation patterns—a mechanism that is called *de novo* methylation—that characterize embryo development, in particular (Chen and Li, 2004).

The maintenance of methylation is crucial for ensuring the continuity of the structural and functional identities of somatic cells throughout cell division. During the S phase of the cell cycle, DNMT1 reaches hemimethylated CpGs with the aid of ubiquitin-like with PHD and RING finger domains 1 (UHRF1) proteins such that each newly synthesized DNA strand can be methylated per its complementary strand. Thus, after each replication, the symmetry of the methylation pattern is restored (Zhang et al., 2011; Wu and Zhang, 2014).

Although methylation patterns are stable, they can be erased by two mechanisms: active and passive demethylation. Passive demethylation represents a failure in maintenance (so-called replication-dependent dilution) and occurs primarily in the absence of functional DNMT1/UHRF1: if the symmetry of methylation is not reestablished, methylation is lost through replications (Smith and Meissner, 2013; Wu and Zhang, 2014).

**Figure 3:**
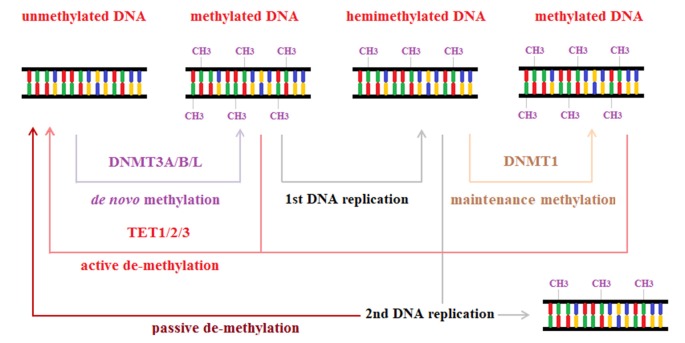
Methylation and demethylation. Methylation is a regulatory process of gene expression, catalyzed by DNA methyltransferase enzymes, owing to the addition of a methyl group to the fifth position of a cytosine. DNA methyltransferases 1 (DNM1) is mainly involved in maintenance methylation that restores symmetric DNA methylation patterns after DNA replication. DNM3A, DNMTB and DNMTL, instead, are involved in the catalytic process that produces *de novo* methylation by adding methyl groups to unmethylated DNA strands. Methylation processes can be reverted by two mechanisms: passive demethylation due to loss of methylation across consecutive DNA replications; active demethylation mediated by ten-eleven translocation (TET) proteins.

Active methylation is mediated by ten-eleven translocation (TET) proteins, which exist as three isoforms: TET1, TET2 and TET3. This subfamily of dioxygenases catalyzes the oxidation of 5mC to hydroxymethylcytosine (5hmC), 5-formylcytosine (5fC) and finally 5-carboxylcytosine (5caC). This conversion is the first step toward complete demethylation through two pathways. In the first mechanism, DNMT1 is less effective toward 5hmC, 5fC and 5acC methylation; thus, the oxidative activity of TET can foster passive dilution. In the second route, 5fC and 5acC can be excised from DNA by thymine DNA glycosylase, and the resulting lesion is promptly repaired through the base excision repair (BER) pathway, generating an unmodified cytosine. Thymine DNA glycosylase and BER are also recruited when 5mC is deaminated to thymine by activation-induced deaminase, particularly in promoter regions during somatic cell reprogramming (Seisenberger et al., 2013; Zhao and Chen, 2013). An overview of methylation and demethylation mechanisms is provided in Figure 3.

#### Methylation and Epigenetic Inheritance

Maintenance and *de novo* methylation and active and passive demethylation are crucial for embryonic development and epigenetic inheritance. Gametes are completely demethylated and are remethylated after fertilization to erase all epigenetic marks that an individual accumulates over his lifespan. However, this resetting process is impeded during early development, perhaps accounting for transgenerational transmission of these epigenetic footprints (van Otterdijk and Michels, 2016).

**Figure 4:**
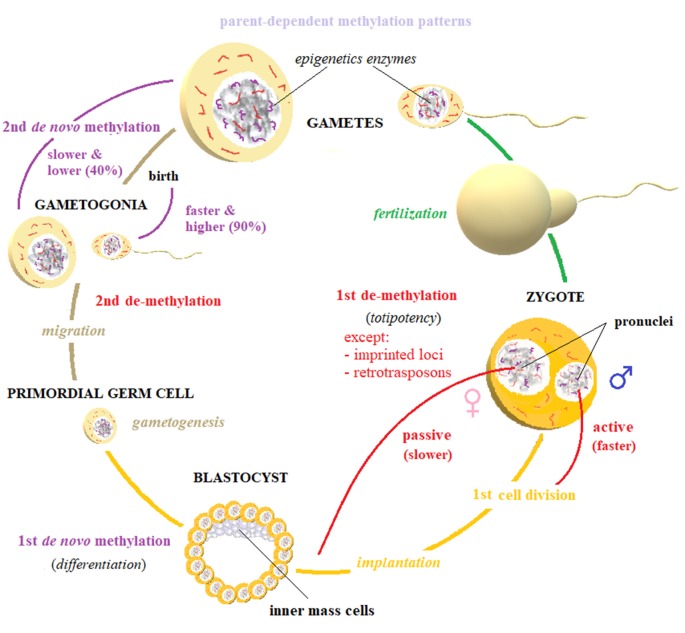
Biomarker reset. The elimination and restoration of methylation markers happen in two steps. A first, active demethylation takes place in parental gametes, right after fertilization. This process is mostly active—and therefore faster: it is completed by the first cell division—for paternally inherited genome, while maternal pronucleus is slowly demethylated by passive diffusion across replications. This first global erasure of methylation marks spares only imprinted loci and some retrotransposons, and it is deemed to establish cellular totipotency. After the implantation of the developing blastocyst, a first *de novo* methylation wave begins, driving the crucial process of cellular differentiation. At the beginning of gametogenesis, when primordial germ cells start to migrate, a second demethylation takes place: gametes’ chromatin is globally demethylated, also including imprinted loci. After sex-determination, gametogonia are remethylated by a second wave of *de novo* methylation, which is higher (90%) and faster (it is mostly complete before birth) for male gametes and slower (40%) and lower (it does not end until puberty) for female gametes. Imprinting patterns are usually reestablished during this phase. The established patterns can be altered by direct or indirect experiences, particularly during gestation and right after birth. These processes depend on the activity of several epigenetic enzymes, among which DNA methyltransferases (DNMTs) and TETs are prominent. The regulation of these processes by non-coding RNA (ncRNA), has also been established.

Elimination and restoration of methylation markers occurs in two steps (Figure 4). Immediately after fertilization, global demethylation is observed that erases methylation marks of the parental gametes through two sex-dependent mechanisms. First, the DNA in paternal pronuclei undergoes rapid, active demethylation that is mediated by TET3 proteins, which spare only imprinting control regions (ICRs) and certain retrotransposons, such as intracisternal A particles. This process takes place at approximately the time of DNA replication and ends before the first cell division is completed. Then, the maternal genome is progressively demethylated through passive demethylation across subsequent cleavage steps (Seisenberger et al., 2013). Consequently, the totipotency of the zygote is established and maintained across the first several cell divisions.

The maternal factor Stella has been suggested to protect the maternal genome and paternal ICRs and intracisternal A particles from active demethylation. These regions undergo H3K9 (a Stella binding site) demethylation. Moreover, inside of the oocyte and zygote, the DNMT1o isoform predominates and is more concentrated in their cytoplasm. In contrast, DNMT1 is the chief isoform in somatic cell nuclei but is scarce in the zygote. These differences in nuclear and cytoplasmic concentrations of DNMT1 isoforms account for global passive demethylation and might explain the maintenance of maternal ICRs (Cardoso and Leonhardt, 1999; Seisenberger et al., 2013). Nevertheless, recent studies suggest that active and passive processes govern the demethylation of the maternal and paternal genomes (van Otterdijk and Michels, 2016). After the implantation of the developing blastocyst, the inner mass cells (IMCs) undergo a wave of* de novo* methylation, which drives their differentiation. This process is mediated by DNMT3 (Chen and Li, 2004; Seisenberger et al., 2013).

A second wave of demethylation is initiated at the outset of gametogenesis: primordial germ cells experience demethylation that starts during their migration and spreads to ICRs (Zhao and Chen, 2013; Wu and Zhang, 2014). After sex determination, gametogonia DNA is remethylated through a second *de novo* methylation step. Notably, male gametes reach methylation levels of 90% before birth, whereas oocytes increase their levels progressively, long after birth and until sex maturation when they decline to approximately 40%; in this phase, imprinted loci are usually restored (Smith and Meissner, 2013; Zhao and Chen, 2013). As argued above, the transmitted patterns can be altered by direct or indirect experiences, particularly during gestation and immediately after birth.

It appears that epigenetic transmission might be possible when the second demethylation step is prevented, as in the case of genomic imprinting, which constitutes the strongest evidence for transgenerational epigenetic inheritance in mammals (van Otterdijk and Michels, 2016). Correct repression of transcription of certain genes is crucial for a good developmental outcome. A glitch during genomic imprinting, for example, can cause severe pathologies, such as Prader–Willi and Angelman syndromes, which are derived from the loss of nonimprinted paternal and maternal genes, respectively (Cassidy et al., 2000).

#### New-Generation Epigenetic Mechanisms

New-generation epigenetic mechanisms also incorporate factors that modify the genetic expression at the translational level, such as alternative splicing, RNA editing, and regulation by ncRNAs (Cooper and Hausman, 2013). Recently, ncRNAs have been implicated in disease development and manifestation and in their epigenetic transmission (Peschansky and Wahlestedt, 2014). ncRNAs are not translated into proteins. Only 2% of RNA is mRNA and becomes a functional and structural component of the cell. The remaining 98%, however, is far from “junk,” as once believed. Many genes are translated into ncRNA (Liu et al., 2013). What do these molecules do? As we will discuss below, many groups (e.g., Amaral et al., 2013; Peschansky and Wahlestedt, 2014) have exhaustively reviewed the functional properties of these newly implicated species in epigenetic regulation and inheritance, highlighting their direct and indirect functions.

#### Non Coding RNA and Epigenetic Regulation

ncRNAs that are less than 200 nucleotides are labeled “short” or “small,” whereas those that exceed this length are defined as “long” (lncRNAs). These two groups can be subdivided, depending on their genomic origin and biogenic activity.

lncRNAs are divided into five subgroups:

- natural antisense transcript (NAT), a complementary sequence to a coding RNA at the same locus (cis-NAT) or a distal genomic locus (trans-NAT).
- long intergenic ncRNA (lincRNA), which is encoded from the introns of intergenic regions (macroRNA or vlincRNA).
- sense overlapping, which is transcribed from the same DNA strand as another transcript.
- sense intronic, originating from the introns of coding genes.
- processed transcript, an RNA transcript that is spliced or polyadenylated.

Whereas NATs primarily regulate the expression of the sense partner transcript, the activities of the other four classes remain unknown, but they are likely to include transcriptional regulation, RNA stability, and the recruitment of protein complexes and other subcellular elements. lncRNAs are usually transcribed and processed similarly to coding mRNAs (Peschansky and Wahlestedt, 2014).

Small RNAs are grouped into five clusters: PIWI-interacting RNAs (piRNAs), endogenous short interfering RNAs (endo-siRNAs), miRNAs (or miRs), transfer-derived RNAs (tDRs or tsRNAs) and small nucleolar RNAs (snoRNAs). PiRNAs are usually composed of 26–30 nucleotides and can silence the transcription of target RNAs, promoting the trimethylation of histone 3 lysine 9 (H3K9me3), a marker of inactive chromatin, by a histone methyltransferase (Luteijn and Ketting, 2013).

The function of endo-siRNAs is not well understood, but it appears to require extensive sequence complementarity to repress genes (Okamura et al., 2008). miRNAs are 20–23-nucleotide segments that usually target mRNAs by complementarity to a 6-nucleotide seed region in the 3′-UTR or 5′-UTR (Vidigal and Ventura, 2012). tsRNAs are derived from the rigid processing of mature precursor tRNAs at the 5’ or 3’ end and have a similar function as miRNA, regulating RNA-silencing activities (Haussecker et al., 2010). snoRNAs are involved in modifications to ribosomal RNAs; but snoRNA is also a miRNA precursor and has a similar function to miRNAs (Ender et al., 2008).

Our understanding of the processes that generate mature small ncRNAs is patchy. Only the biogenesis of miRNAs has been determined. The formation of miRNAs begins in the nucleus with the transcription of a primary miRNA (pri-miRNA) by RNA polymerase II (RNA Pol II). Pri-miRNAs are attacked by the microprocessor complex, composed of RNase III (Drosha) and DGCR8 (Pasha). Drosha cleaves pri-miRNA into a shorter transcript, whereas Pasha stabilizes the interaction between Drosha and pri-mRNA. This catalytic event produces a stem-loop structure, the precursor miRNA (pre-miRNA). The pre-miRNA is then exported to the cytoplasm by Ran-GTP, which energizes the transport system, and exportin-5 (EXP5), which interacts directly with the stem-loop structure. Here, the pre-miRNA associates with Dicer (another RNase III), which cleaves it into two molecules of approximately 22 nucleotides: guide strand (or mature miRNA) and passenger strand (or miRNA*). These two species are then loaded into argonaute (Ago) proteins, which select the mature miRNA (while miRNA* is degraded) and deliver it to the RNA-induced silencing complex, through which it arrives at its targets, destabilizing mRNA and inhibiting transcription (Blahna and Hata, 2012).

In general, lncRNAs and small RNAs intervene in several regulatory processes in nuclear architecture, chromatin regulation, transcriptional regulation and RNA processing (Amaral et al., 2013; Quinn and Chang, 2016). lncRNAs regulate epigenetics by remodeling chromatin structure, whereas the function of miRNAs in epigenetic processes is linked to their direct or indirect regulation of DNMT expression during embryonic development and in somatic cells (Peschansky and Wahlestedt, 2014).

ncRNAs can be epigenetic targets and epigenetic effectors. Their genetic loci can be subject to epigenetic regulation, like protein-coding genes, becoming susceptible of environmental influences; further, they govern gene expression (Peschansky and Wahlestedt, 2014; Szyf, 2015). This dual nature of ncRNAs implicates them as “change amplifiers.” In this sense, ncRNAs are similar to transcription factors.

#### Small RNAs and Epigenetic Inheritance

Among all classes of small RNAs, miRNAs are the most frequently cited with regard to epigenetic inheritance. Recently, the miRNA expression patterns in placental (Gu et al., 2013; Maccani et al., 2013) and germ cells (Soni et al., 2013; Rodgers et al., 2015) have been implicated in fetal programming, and increasing evidence is considering the function of miRNAs in mediating transgenerational epigenetic inheritance of stress responsivity (Babenko et al., 2015; Fraser and Lin, 2016; Pang et al., 2017; Yeshurun and Hannan, 2018).

As discussed, fetal programming alone does not account for epigenetic transmission, unless we include the effect of previous environmental factors (i.e., AIE) in its definition. As pointed out by Bohacek and Mansuy (2015), germ cell reprogramming could be a key mechanism of transgenerational epigenetic inheritance. Notably, miRNAs control *de novo* DNA methylation by regulating transcriptional repressors (Sinkkonen et al., 2008). Epigenetic changes in germ cells arise and are maintained throughout methylation and acetylation, but miRNAs, particularly those in sperm, appear to have important functions (e.g., Bohacek and Mansuy, 2015; Rodgers et al., 2015; Fraser and Lin, 2016; Pang et al., 2017; Yeshurun and Hannan, 2018).

Conversely, global suppression of miRNA (paired with the functional predominance of endo-siRNAs) has been observed in mature oocytes and during early embryonic development (Ma et al., 2010; Suh et al., 2010). Consistent with these data, oocytes lack DGCR8 (Pasha), which is necessary for miRNA but not endo-siRNA pathways (Ma et al., 2010). miRNAs could be important mediators of placental development through their regulation of genetic expression (Babenko et al., 2015). Their function in the latter phases of zygote development remains unknown, but as we will discuss, there is evidence of the role of miRNAs in the regulation of oocyte function (Tang et al., 2007; Soni et al., 2013). piRNAs are another class of small RNAs that are important in epigenetic inheritance and are highly expressed in sperm and oocytes; tsRNAs, which are enriched in mature mouse sperm, are critical in epigenetic inheritance (Peng et al., 2012; Roovers et al., 2015; Chen Q. et al., 2016; Sharma et al., 2016). However, much work is needed to determine their functions.

### Mechanisms of Epigenetic Inheritance: An Overview

Epigenetic inheritance has been suggested to be governed by the crosstalk between canonical epigenetic mechanisms (primarily methylation) and the regulation of gene expression by ncRNAs at the translational and transcriptional levels, as proposed by several groups (e.g., van Otterdijk and Michels, 2016; Houri-Zeevi and Rechavi, 2017; Pang et al., 2017; Yeshurun and Hannan, 2018). Although there is no direct evidence of the exact mechanisms that are involved, some hypotheses can be introduced.

NcRNAs might mediate the establishment of new patterns of gene expression by regulating DNMT1 and TET in adult somatic cells (DE). Following fertilization, synchronous alterations to the intrauterine environment could define new expression patterns (WIE), particularly through the activities of small ncRNAs on DNMT1, DNMT3A/B/L and TET, interfering with the maintenance of preexisting epigenetic hallmarks. Depending on when an environmental change occurs, the influence on the offspring might depend on the offspring’s sex and materialize using a sex-specific cluster of enzymes (see Figure 4).

The most notable—albeit more obscure and less extensively studied—function of ncRNAs could be to establish the intrauterine environment and gametes before conception, producing new, stable epigenetic marks, such as methylation, that are stably maintained at least across one generation (AIE). Further, the direct transmission of ncRNAs through paternal sperm or fluids and maternal germ cells could intervene in setting epigenetic patterns. Conversely, the presence or absence of molecular tags (such as UHRF1) could influence the expression of crucial ncRNAs during the first or second stage of demethylation, also sex-dependently (Figure 4). These models are only some of the hypotheses that our current understanding allows, and surely overlooks other less well-understood processes, such as histone modification and retention, DNA hydroxymethylation, and chromatin remodeling. These mechanisms are suggested to be relevant to epigenetic inheritance and subject to some form of regulation by ncRNAs, necessitating further evidence of their implication (Babenko et al., 2015; Bohacek and Mansuy, 2015; van Otterdijk and Michels, 2016).

## Methodological Matters: Maternal vs. Paternal Contribution

As pointed out by many groups, (Dunn et al., 2011; Gapp et al., 2014; Babenko et al., 2015), the maternal epigenetic contribution has been studied primarily during pregnancy, whereas the paternal input has been increasingly attributed to sperm, which has recently been demonstrated to be a crucial purveyor of epigenetic information. This model is consistent with the growing body of literature above (see Yeshurun and Hannan, 2018 for an exhaustive review). These effects have been studied widely in terms of the transmission of stress sensitivity in animal and human models (see above). For example, in rodents, stressing the mother during pregnancy and the father before mating can effect alterations in stress sensitivity in the offspring, manifesting at the molecular and behavioral levels (Dunn et al., 2011).

Paternal experiences can induce changes in the sperm that impact, for example, the HPA axis in progeny, their cognitive abilities, and their cellular and molecular processes (Yeshurun and Hannan, 2018). Many authors posit that this type of epigenetic transmission of environmental information determines the miRNA composition of paternal sperm, which is sensitive to environmental changes (e.g., Rodgers et al., 2015; Pang et al., 2017; Yeshurun and Hannan, 2018). According to some groups, miRNAs mediate this form of transgenerational communication, based on their ability to regulate the remethylation that occurs during gamete maturation and fertilization (Sinkkonen et al., 2008; Rodgers et al., 2015; van Otterdijk and Michels, 2016; Yeshurun and Hannan, 2018).

As discussed, paternal influence is not limited to sperm: it can contribute during pregnancy as a stimulus that influences the maternal environment of the fetus (Todrank et al., 2011) and, at least in our species, as a caregiver (Braun and Champagne, 2014). Similarly, oocytes could transmit epigenetic marks of maternal experiences that occur before pregnancy. We should consider that the germ cells in both sexes can be modified epigenetically during fetal development and after birth, throughout life (despite little evidence to support this hypothesis concerning oocytes). Moreover, certain miRNAs (primarily Let-7, miR-30 and miR-16 but also miR-34a) are maternally inherited and depend on maternal miRNA-processing machinery (Tang et al., 2007; Soni et al., 2013). The best evidence of the importance of maternal miRNA, however, is the discovery of a paternally imprinted 14q32 domain, which allows the exclusive maternal expression of approximately 40 miRNAs (Seitz et al., 2004).

The first studies on epigenetic forms of transmission focused on the effects of maternal care on the early stages of life, later considering nongenetic forms of developmental programming of fetal development during pregnancy. A practical problem arose, however: because mothers carry their children for 9 months and then care for them, it was difficult to distinguish between pre-, peri- and postnatal epigenetic effects. Thus, several groups concluded that the paternal contribution should be considered. In many species, the only contribution of males is their sperm, which does not interfere with the gestational and postnatal periods.

This approach has been useful in demonstrating epigenetic inheritance, but it does not allow one to frame the entire landscape of mechanisms of epigenetic transmission: excluding maternal pregestational function because it is intractable for study fails to demonstrate that it does not exist or that it is irrelevant. Most of the literature has focused on the paternal role in mediating AIE (see Yeshurun and Hannan, 2018), whereas maternal function has been neglected. The drawback of many models of epigenetic inheritance is that they do not allow one to distinguish and define paternal and maternal contributions simultaneously for every effector that mediates the transmission of a certain property, such as stress reactivity. Stress vulnerability could result from the co-occurrence of maternal and paternal factors or show maternal or paternal preference, depending on the effector (e.g., which miRNA or group of miRNAs). Further, the prevalence of maternal and paternal contributions could depend on environmental conditions that could bring about, for example, paternal prevalence when the father is stressed or the predominance of maternal contribution under baseline conditions.

### Methodological Insights and Technical Niceties

Bohacek and Mansuy (2015) have suggested methodological practices that could mitigate the effects of the intervenient factors above. For example, artificial insemination or *in vitro* fertilization (IVF) should allow one to exclude the effects of seminal fluid and interactions during mating. The disadvantage of these techniques, however, is that they require superovulation (a fertilization procedure that increases the number of oocytes that are produced) and the use of culture for IVF and chemical manipulation, which could alter ecological epigenetic programs.

**Table 2:** Some useful techniques that can be used to study and control for some crucial developmental variables.

Technique	*Controls for…*
*In vitro* fertilization	*Seminal fluids and interactions during mating*
Embryo transfer	*Intrauterine environment*
Cross-fostering	*Maternal care*
Direct injection	*Contingent action of a specific effector*
Knock out, knock down, over expression	*Effects of gene expression modulation*
Knock out + cross breeding	*Genomic imprinting*

Intrauterine and maternal care could be controlled for through embryo transfer and crossfostering, respectively. The function of a specific effector, independent of its parental origin, could be tested by injecting molecules directly into a zygote (Bohacek and Mansuy, 2015) or germ cells. Genetic expression in the embryo can be manipulated using KO, OE and knockdown (KD) models. When the gene of interest is missing from birth, several changes are developmentally established, which are nonspecific and stable. The inconvenience of these models is that the effects of manipulating a certain gene result from a series of functional and structural adaptations throughout development. An alternative solution is to use conditional models and other genetic engineering techniques, as reviewed by Issler and Chen (2015). By combining a KO model with a double crossbreeding procedure, once behavioral profiles have been defined for the WT and KO lines, it should be possible to determine whether a certain gene is subject to genomic imprinting, simply by observing the offspring phenotypes; if it is, two divergent behavioral tendencies should be observed. However, imprinting is not an all-or-nothing phenomenon, and ambiguous results could be clarified with other techniques that measure allelic expression directly (see Rienecker et al., 2016 for a review). The discussed techniques are summarized in Table 2.

### Defining the Spacetime of Epigenetic Inheritance: Ideal Models

As reported above, several experiments have been conducted to demonstrate the existence of epigenetic inheritance. The results remain incomplete and sometimes conflicting, perhaps because only one route of transmission is usually considered at a time (e.g., maternal stress during pregnancy, paternal stress before mating). Moreover, the same type of event can occur in disparate moments and contexts, targeting subsequent generation through different routes.

This possibility implies that it would be better to apply several types of environmental conditions on all possible levels. For example, male and female mice could be stressed immediately prior to or long before fertilization—mildly or robustly and acutely or chronically—but also during gestation or after delivery (the latter two with regard to mothers only). It would then be interesting to study how a certain transmitted vulnerability interacts with an environmental condition that is similar to the causative factor throughout the offspring’s life. This approach is consistent with the model that, as in genetic inheritance, epigenetic inheritance can mediate the transmission of vulnerability (considered a type of epigenetic diathesis), which could remain silent and unexpressed unless—or until, depending on one’s degree of fatalism—certain environmental events take place (Godfrey et al., 2007). Once epigenetic inheritance has been detected, the next crucial step is to determine the underlying molecular mechanisms.

The specific spacetime of an action of an epigenetic effector that is suspected to mediate transgenerational epigenetic transmission (for example, a miRNA) should be identified using the following experimental design. In a murine model, WT and manipulated (M)—i.e., KO, OE, or KD of the gene that encodes the epigenetic effector—oocytes could be fertilized with WT or M sperm in all possible combinations through IVF or natural breeding that is paired with embryo explants and implantation. The four possible types of zygotes that are produced could be implanted in WT or M dams—the latter of which allows one to control the effects of the intrauterine environment (including the placenta).

**Figure 5:**
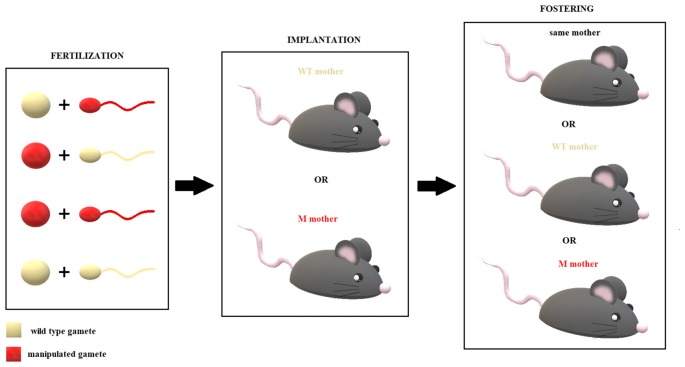
From *in vitro* fertilization (IVF) to fostering. Here, we schematize the suggested ideal model that could help define with great precision the spacetime of a given epigenetic factor’s action. Once its role in fetal programming has been established, investigating its possible play in transgenerational epigenetic inheritance processes might be easier. See the text for more details.

Once they are born, the pups should be raised by their mother or a WT or M foster mother to control for the effects of maternal behavior (Figure 5). Although a fostering experience has been demonstrated to constitute a relevant stressful experience that can alter the developmental trajectory—primarily if it is repeated (Ventura et al., 2012; Di Segni et al., 2016)—it likely remains the most cogent means of controlling for the effects of maternal care. Observing the behavioral outcomes of the various levels of manipulation could allow one to identify the point at which the lack of a certain transcriptional product has relevant consequences and thus when and where its activity is necessary.

Conditional models are preferred when defining the weight of a specific effector in a specific place and time (e.g., during paternal or maternal gametogenesis, zygote formation, the third week of gestation throughout the placenta, right after birth). In contrast, developmental models should allow one to observe the final, complex outcome of a certain alteration of a gene (such as polymorphisms and genes that encode epigenetic elements) in a more complex, systemic manner. The latter approach is not conducive to gaining a precise understanding of mechanisms but still has ecological value that cannot be ignored.

Another noteworthy issue concerns whether to use IVF or natural breeding, followed by embryo extraction and implantation. IVF requires superovulation and the use of an artificial culture, which could alter the programing of gametes (Bohacek and Mansuy, 2015). The use of natural breeding, conversely, fails to control for the effects of the manipulation of male and female reproductive fluids (Bohacek and Mansuy, 2015), warranting further comparison with offspring that result from natural breeding.

These considerations are pivotal to correctly interpret data, despite the manipulation of a factor and the breeding procedure (e.g., conditional vs. developmental and artificial vs. natural). Moreover, the proposed model is only theoretical and does not impose its complete application, although it would likely produce the strongest evidence possible, whatever results emerge. Once the activity of a certain effector has been described, a more specific molecular analysis can be conducted to link the steps of the underlying mechanism of the specific process of epigenetic inheritance.

## Conclusions and Future Perspectives

In this review article, we have introduced the concept of epigenetics, defining its spatial and temporal properties, allowing us to distinguish between types of epigenetics: a direct form of epigenetics (DE) and two forms of indirect epigenetics—within (WIE) and across (AIE). We have organized the main body of epigenetic evidence according to these three categories and focused on the latter (AIE), referring to it as a more rapid means of transmitting information across generations—compared with genetic inheritance—that guides human evolution in a Lamarckian (i.e., experience-dependent) manner. We have thus defined epigenetic inheritance in terms of AIE and illustrated the putative molecular mechanisms of this phenomenon.

Finally, we have discussed the main methodological matters regarding the study of epigenetic inheritance and have suggested strategies to solve some of the most compelling technical and theoretical problems that plague this field. The experimental models that we have proposed are inapplicable to human research, for obvious ethical reasons, but if we detail the mechanisms that underlie epigenetic inheritance, thus isolating key effectors to examine, we could study the “natural experiments” that we have (and probably will) occasionally encountered in history. There is no doubt that translational research could benefit from this scientific effort. Epigenetic inheritance, when maladaptive, can have a silent, unseen, but dramatic impact on health, perpetrating detrimental adaptations across generations.

The peculiar nature of epigenetics could allow us to intervene at various levels synchronously, thus applying more effective synergistic activity against complex diseases, which have not been able to be properly understood or approached until now—particularly on the molecular level. We could prevent malicious epigenetic forms of inheritance, evaluating the quality of germ cells in high-risk cases and eventually administering pharmacological treatments that target specific epigenetic mechanisms, as recently suggested by Pang et al. (2017). Germline and somatic cells have been studied as putative targets for genetic therapy to prevent the evolution and transmission of several human pathologies (Baltimore et al., 2015), even those that are caused by abnormalities in maternal mitochondrial DNA (Hyslop et al., 2016).

Thus, there is no reason why a similar therapeutic approach should be overlooked for epigenetic abnormalities that affect an individual at early age and even during fetal development.

The environment is another level that confirms its well-established function as an epigenetic regulator and is also thus a potentially invaluable therapeutic “tool” (Maccari et al., 2017). To strengthen the therapeutic power of the environment, paradoxically, we must understand the specific mechanisms that are altered by epigenetic adaptations following certain experiences. Yeshurun and Hannan (2018) have suggested a therapeutic/preventive approach, called “enviromimetics,” that aims to ameliorate paternal psychophysical conditions before conception to revert or prevent epigenetic alterations in sperm, thus reducing the transgenerational impact of stress.

Finally, we can use epigenetic biomarkers for diagnostic purposes and trace their levels over time to evaluate and monitor therapeutic efficacy (O’Connor et al., 2016). Notably, several peripheral biomarkers have been defined to assess placental dysfunction and other general abnormalities during pregnancy (Maccani et al., 2013; Morales-Prieto et al., 2014). Conversely, it would be helpful to monitor the observable, phenomenological patterns (e.g., behavior for psychopathologies) that specify the underlying epigenetic mechanisms that should be treated—to understand “where and what” to look.

Attaining this ideal therapeutic power will require new studies on AIE—particularly on the gap between two generations. These studies could ensure greater “spacetime resolution” of such a complex phenomenon, thus facilitating the development of a prompt and effective intervention. Although we have detailed how epigenetic factors can lead to many pathologies, we must be reminded that they are usually crucial in all of the adaptive processes that ensure the survival of the individual and species (van Otterdijk and Michels, 2016). For this reason, the decision to interfere with their activity should be strongly supported by a profound understanding of the specific case in question and applied with great caution.

## Author Contributions

IL conceived the general theoretical framework, collected most of the bibliography, wrote the first draft of the article and designed and drew the figures. Both authors developed, refined and carefully reviewed the final version of the article.

## Conflict of Interest Statement

The authors declare that the research was conducted in the absence of any commercial or financial relationships that could be construed as a potential conflict of interest.
